# Comprehensive Insight into Microcystin-Degrading Mechanism of *Sphingopyxis* sp. m6 Based on Mlr Enzymes

**DOI:** 10.3390/toxins17090446

**Published:** 2025-09-05

**Authors:** Qin Ding, Tongtong Liu, Zhuoxiao Li, Rongli Sun, Juan Zhang, Lihong Yin, Yuepu Pu

**Affiliations:** Key Laboratory of Environmental Medicine Engineering, Ministry of Education, School of Public Health, Southeast University, Nanjing 210009, China; dingqin@seu.edu.cn (Q.D.); yppu@seu.edu.cn (Y.P.)

**Keywords:** Microcystin, *Sphingopyxis*, Mlr enzyme, degradation mechanism, gene knockout

## Abstract

Bacterial degradation is one important Microcystin (MC) removal method in the natural environment. The traditional MC-degrading pathway was proposed based on the functions of individual recombinant Mlr enzymes and the structures of the main MC-degrading products. However, the actual MC-degrading mechanism by Mlr enzymes in wild-type bacteria remains unclear. In this study, bioinformatic analysis, heterologous expression, and knockout mutation were performed to elaborate the MC-degrading mechanism by Mlr enzymes in *Sphingopyxis* sp. m6. The results showed that *mlr* gene cluster was initially acquired by horizontal gene transfer, followed by vertical inheritance within *Alphaproteobacteria*. Mlr enzymes exhibit distinct subcellular localizations and possess diverse conserved catalytic domains. The enzymatic cascade MlrA/MlrB/MlrC sequentially cleaves Microcystin-LR (MC-LR) via Adda-Arg, Ala-Leu, and Adda-Glu bonds, generating characteristic intermediates (linearized MC-LR, tetrapeptide, and Adda). Notably, recombinant MlrC demonstrated dual-targeting degrading capability (linearized MC-LR and tetrapeptide), while tetrapeptide specificity in endogenous processing of *Sphingopyxis* sp. m6. Marker-free knockout mutants of *mlr* genes were first constructed in MC-degrading bacteria, unveiling that *mlrA* was indispensable in initial MC cleavage, whereas *mlrB*/*mlrC*/*mlrD* displayed functional compensation through other enzymes with similar functions. This study promotes the mechanistic understanding of MC bacterial degradation and offers a theoretical basis for a bioremediation strategy targeting cyanotoxin pollution.

## 1. Introduction

Cyanobacterial harmful algal blooms (cHABs) frequently outbreak in eutrophic freshwater worldwide, critically endangering aquatic ecosystems and human health through excessive cyanobacterial proliferation and associated cyanotoxin release [1,2,3]. Among these cyanotoxins, Microcystins (MCs) are a group of cyclic heptapeptides predominantly synthesized and released by *Microcystis*, *Anabaena*, and *Planktothrix* genera [4]. MCs demonstrate ubiquitous environmental distribution across aquatic, terrestrial, and atmospheric matrices, with bioaccumulation potential through trophic transfer [5,6,7,8]. Their pervasive contamination raises global concerns due to the potent hepatotoxicity and potential carcinogenicity [2,9,10,11,12]. Among the more than 200 congeners, Microcystin-LR (MC-LR) is one of the most abundant and toxic hepatotoxins with the structure of cyclo-(Ala-Leu-MeAsp-Arg-Adda-Glu-Mdha), where Adda is 3-amino-9-methoxy-2,6,8-trimethyl-10-phenyl-deca-4,6-dienoic acid [13]. Owing to their stable cyclic structure, MCs exhibit resistance to conventional water treatment processes and thermal degradation, presenting a significant risk to drinking water safety and necessitating the advancement of effective detoxification methodologies [14,15]. Biodegradation, emerging as an ecologically sustainable remediation approach, has multiple advantages in the elimination of MCs and restoration of contaminated aquatic environments [16,17]. To date, multiple studies have demonstrated that both aerobic and anaerobic microbial communities from MCs-contaminated environmental samples possess the ability to degrade MCs [18,19,20,21].

Despite MC-degrading bacteria are prevalent in the natural environment, only dozens of strains have been found with a wide variation in degradation capacity [13,22]. Most of them are *Sphingomonas*, *Sphingopyxis*, and *Novosphingobium*, belonging to the *Sphingomonadaceae* family and *Alphaproteobacteria* class [16]. The canonical pathway proposes that these bacteria utilize Mlr enzymes (MlrA, MlrB, MlrC, and MlrD enzymes) encoded by *mlr* gene cluster (*mlrA*, *mlrB*, *mlrC,* and *mlrD* genes, respectively) to perform sequential enzymatic MCs degradation [23,24]. More specifically for MC-LR, MlrA initiates the degradation process by hydrolyzing the cyclic MC-LR into linearized MC-LR (Adda-Glu-Mdha-Ala-Leu-MeAsp-Arg). Subsequently, MlrB cleaves the linearized MC-LR into a tetrapeptide (Adda-Glu-Mdha-Ala). MlrC could degrade both the linearized MC-LR and tetrapeptide into Adda [25,26]. MlrD is hypothesized as an oligopeptide transporter to the transmembrane transport of MC-LR and its degradation products [24,27]. However, the current understanding of MC-degrading mechanisms by Mlr enzymes remains fragmented. First, the degrading functions of Mlr enzymes were determined in vitro through the heterologous expression of *mlr* genes. Affected by the intracellular environment, the actual degrading activities of Mlr enzymes may be different in wild-type MC-degrading bacteria. Then, the traditional MC-degrading pathway was proposed according to the chemical structure of the above three degrading products. The spatial position of enzymes in wild-type MC-degrading bacteria and other degrading products was not considered. Furthermore, new evidence suggests that non-*mlr* MC-degrading mechanisms may synergize with *mlr*-dependent hydrolysis, contributing to the bacterial degradation of MCs [18,28]. According to the above information, there are knowledge gaps regarding how Mlr enzymes are involved in the MC-LR degradation in with-type bacteria.

An indigenous efficient MC-degrading *Sphingopyxis* sp. m6 (Genbank, accession number: MF535105) was isolated from Lake Taihu previously, which harbors the complete *mlr* gene cluster (Genbank, accession number: MK179284–MK179287) [29,30,31]. However, the MC-degrading mechanism of *Sphingopyxis* sp. m6 was unknown. In this study, we will investigate the degradation mechanism of MC-LR by *Sphingopyxis* sp. m6 comprehensively, focusing on (i) the character of *mlr* genes/Mlr enzymes, (ii) the function of Mlr enzymes involved in MC-LR degradation, and (iii) the specific MC-degrading process by Mlr enzymes using bioinformatics, heterologous expression, and knockout mutation analysis. The results will promote a mechanistic understanding of MCs bacterial degradation and provide potential strategies for aquatic ecosystem restoration and freshwater safety management.

## 2. Results

### 2.1. Phylogenetic Analysis of mlr Genes in Sphingopyxis sp. m6

**Figure 1:**
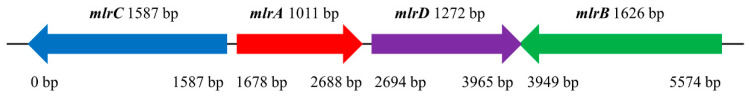
Distribution of the *mlr* gene cluster on the genomic island of *Sphingopyxis* sp. m6.

Previous studies and the expression level of the *mlr* gene cluster suggested that *mlrA*, *mlrB*, *mlrC*, and *mlrD* genes may be involved in the degradation of MC-LR in *Sphingopyxis* sp. m6 [29,32,33]. Based on the bacterial genome and predicted ORFs, the distribution of the *mlr* gene cluster within the genome of *Sphingopyxis* sp. m6 was analyzed. The *mlr* gene cluster in *Sphingopyxis* sp. m6 exhibited atypical organization (*mlrC*-*mlrA*-*mlrD*-*mlrB*) within a 77.44 kb genomic island and divergent transcriptional orientations (*mlrA*/*mlrD* vs. *mlrB*/*mlrC*) (Figure 1).

The *mlrA* gene of *Sphingopyxis* sp. m6 is closest to that of *Sphingomonas* sp. USTB-05, *Sphingopyxis* sp. MB-E, and *Sphingopyxis* sp. YF1 genome. The phylogenetic relationships are centered in the *Alphaproteobacteria* order, especially within the *Sphingomonadaceae* family (Figure S2). The *mlrB*, *mlrC*, and *mlrD* genes of *Sphingopyxis* sp. m6 show congruent clustering patterns to *mlrA* (Figures S3–S5). This conserved phylogenies of *mlrA*, *mlrB*, *mlrC*, and *mlrD* genes within the *Sphingomonadaceae* and *Alphaproteobacteria* suggest evolutionary constraints on operon integrity (Figures S2–S5). Significant differences in GC content between the *mlr* gene cluster (57.6–60.0%) and bacterial genome (66.57%) indicated that *Sphingopyxis* sp. m6 may acquire *mlr* gene cluster by horizontal gene transfer (HGT) followed by vertical inheritance.

### 2.2. Characters of Mlr Enzymes in Sphingopyxis sp. m6

The structural properties of MC-degrading enzymes, including signal peptide, transmembrane (TM) protein, theoretical isoelectric point (pI), molecular weight (MW), subcellular localization, and conserved protein domain family of Mlr enzymes, are listed in Table 1. TMHMM Server v.2.0 (https://services.healthtech.dtu.dk/service.php?TMHMM-2.0, accessed on 2 July 2025) and DeepTMHMM (https://dtu.biolib.com/DeepTMHMM, accessed on 2 July 2025) analysis revealed that MC-degrading enzymes MlrA and MlrD are transmembrane proteins (Figures S6 and S7). Additionally, the predicted probability of MlrA having a signal peptide is 56.90%, while MlrB was is 95.20% (Figures S8 and S9).

By analyzing the conserved amino acid sequences of Mlr enzymes, we found that MlrA is classified as an endoplasmic reticulum metalloproteinase, a member of CAAX protease and bacteriocin-processing enzyme (CPBP) family, which corresponds to the Abi protein family (CAAX amino-terminal protease) in the protein families database (pfam) and contains the Abi domain. Abi domain is widely recognized for its possible protein membrane anchoring in eukaryotes and the bacteriocin immune function in prokaryotes [34]. The highly conserved amino acid residues of Abi domain are Glu^172^, Trp^176^, Trp^201^, His^205^, His^260^, and Asn^264^ (Figure S8). MlrB is classified as a serine hydrolase domain-containing protein that can have both hydrolase and transferase activities. The amino acid sequence from position 39–349 is similar to the domain of β-lactamase and penicillin-binding protein transpeptidase, with Ser^77^ and Lys^80^ as the conserved sites (Figure S9). MlrC is categorized as an M81 family metalloprotease, containing the DUF1485 domain, with amino acid residues Glu^56^, His^150^, Asp^184^, His^186^, and His^208^ distributed around Zn^2+^, forming the catalytic active center. MlrD contains the PTR2 domain and belongs to the Major Facilitator Superfamily (MFS).

### 2.3. Heterologous Expression and Enzymatic Activity of Mlr Enzymes

**Figure 2:**
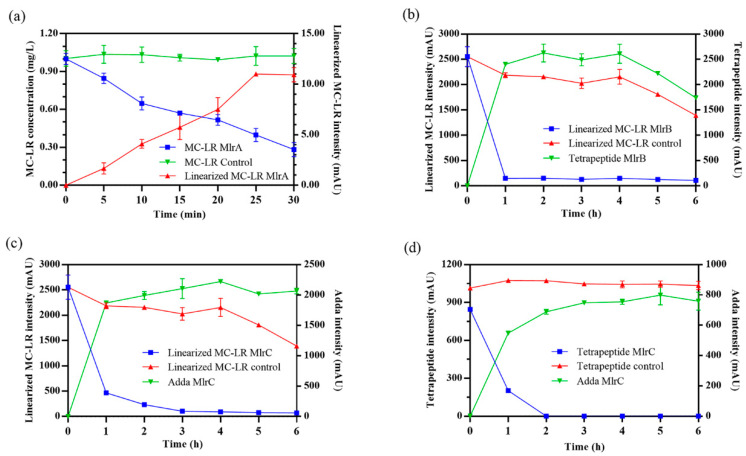
Degradation process of Microcystin-LR (MC-LR) and its main products by MlrA, MlrB, and MlrC. (**a**) MC-LR was degraded by MlrA. (**b**) Linearized MC-LR was degraded by MlrB. Linearized MC-LR (**c**) and tetrapeptide (**d**) were degraded by MlrC. Heat-inactivated enzymes were used in control groups.

Heterologous expression and knockout mutagenesis are important methods to verify the function of the target gene in microbiology [35]. In this study, heterologous expression of *mlrA*, *mlrB*, and *mlrC* genes was performed, and the recombinant Mlr enzymes (MlrA, MlrB, and MlrC) were further purified referred to our previous research [36]. Then, the degrading ability of the recombinant Mlr enzymes to MC-LR and its main products was tested. Through the qualitative and quantitative analysis of MC-LR and its main degrading products, their degrading ability is presented in Figure 2 and Table S1.

**Figure 3:**
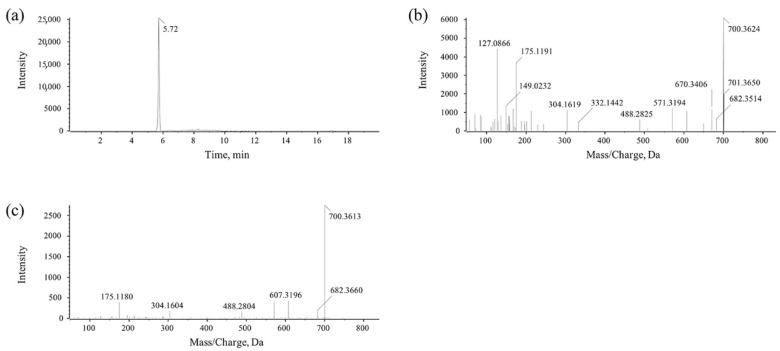
UPLC-MS/MS detection for the hexapeptide compound. (**a**) Chromatogram of hexapeptide. (**b**) Parent ion of hexapeptide. (**c**) Daughter ions of hexapeptide.

**Figure 4:**
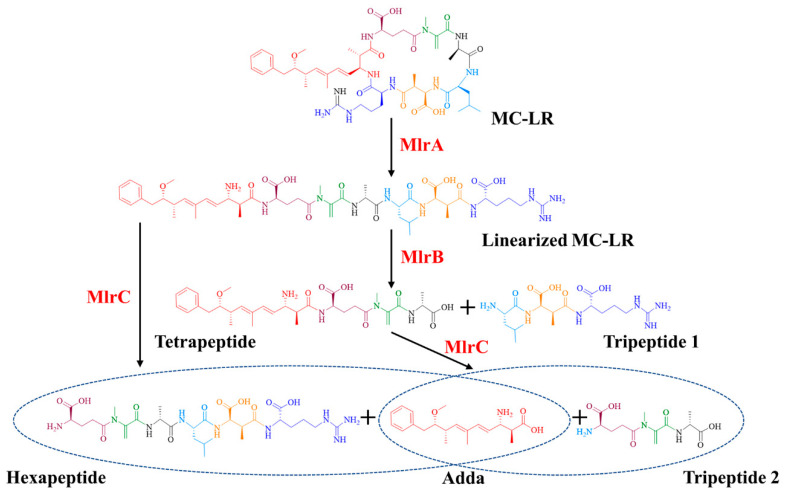
The degrading process of MC-LR by recombinant Mlr enzymes from *Sphingopyxis* sp. m6.

MlrA can hydrolyze cyclic MC-LR at the Adda-Arg bond to generate linearized MC-LR (Adda-Glu-Mdha-Ala-Leu-MeAsp-Arg) with an average degradation rate of 1.44 mg/L/h within 30 min (Figure 2a). MlrB is capable of hydrolyzing linearized MC-LR at the Ala-Leu bond generating a tetrapeptide (Adda-Glu-Mdha-Ala) and a tripeptide compound (tripeptide 1, Leu-MeAsp-Arg) (Figure 2b). The degradation ratio of linearized MC-LR reached 94.16% and the intensity of tetrapeptide 1 increased rapidly within the first hour. MlrC can degrade linearized MC-LR into Adda and a hexapeptide compound (Glu-Mdha-Ala-Leu-MeAsp-Arg) at the Adda-Glu bond (Figure 2c and Figure 3). MlrC achieved a degradation ratio of 95.89% for linearized MC-LR within 3 h and a significant 81.73% reduction occurred within the first hour. Concurrently, the content of Adda rise rapidly in the first hour and subsequently exhibited a gradual increase. Meanwhile, MlrC can cleave the tetrapeptide at the Adda-Glu bond generating Adda and another tripeptide compound (tripeptide 2, Glu-Mdha-Ala) (Figure 2d). The degradation ratio of the tetrapeptide by MlrC reaches 76.04% within the first hour. The specific degrading process of MC-LR by MlrA, MlrB, and MlrC is presented in Figure 4.

### 2.4. Knockout Mutant of mlr Genes and Degrading Function Determination

The pLP12 suicide plasmid containing the upstream and downstream homologous arms underwent two rounds of homologous recombination with the bacterial genome of *Sphingopyxis* sp. m6 to replace the target genes. The CM resistance gene served as a selection marker. Through the multiple selection, the mutants of *mlrA*, *mlrB*, *mlrC*, and *mlrD* genes in wild-type *Sphingopyxis* sp. m6 were successfully constructed, designated m6-ΔmlrA, m6-ΔmlrB, m6-ΔmlrC, and m6-ΔmlrD, respectively.

**Figure 5:**
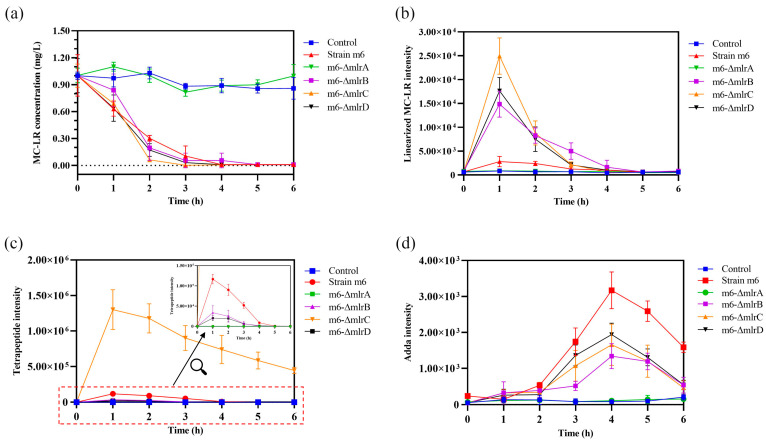
MC-LR degradation by *mlr* knockout mutants and the change in degrading products. (**a**) MC-LR. (**b**) Linearized MC-LR. (**c**) Tetrapeptide. (**d**) Adda.

The MC-LR degrading abilities of *Sphingopyxis* sp. m6 and *mlr* knockout mutants were analyzed to identify the function of *mlr* genes in MCs degradation. As shown in Figure 5a, *Sphingopyxis* sp. m6, m6-ΔmlrB, m6-ΔmlrC, and m6-ΔmlrD were capable of degrading MC-LR. There was no statistical difference in their degrading abilities, and the degrading curves were similar. This suggests that *mlrB*, *mlrC*, and *mlrD* genes are not involved in the first step of MC-LR degradation, but rather in the degradation of intermediate products. However, the m6-ΔmlrA mutant strain was unable to degrade MC-LR, indicating that *mlrA* is essential to the first step of MC-LR degradation. Combined with the function of recombinant MlrA, we hypothesized that MlrA may be the only enzyme hydrolyzing cyclic MC-LR to linearized MC-LR in *Sphingopyxis* sp. m6.

### 2.5. Degradation Process of MC-LR by mlr Knockout Mutants

The degrading products of MC-LR by *mlr* knockout mutants (m6-ΔmlrB, m6-ΔmlrC, and m6-ΔmlrD) were identical to those by *Sphingopyxis* sp. m6, including linearized MC-LR, tetrapeptide, and Adda [29]. However, no degradation product was detected in mutant m6-ΔmlrA and control groups. These results suggest that, while *mlrA* is indispensable in *Sphingopyxis* sp. m6, *mlrB*, *mlrC*, and *mlrD* genes may have functionally similar counterparts in MC-LR degradation.

The dynamics of main products during MC-LR degradation by *mlr* knockout mutants are shown in Figure 5b–d. The concentration of linearized MC-LR in *Sphingopyxis* sp. m6, m6-ΔmlrB, m6-ΔmlrC, and m6-ΔmlrD groups initially increased to a maximum within 1 h, followed by a decrease to the minimum value within 5 h (Figure 5b). Compared to *Sphingopyxis* sp. m6, the maximum intensity of linearized MC-LR in m6-ΔmlrB, m6-ΔmlrC, and m6-ΔmlrD groups increased by 4.30-fold, 7.90-fold, and 5.29-fold, respectively. The deletion of *mlrB* blocked the degradation of linearized MC-LR into a tetrapeptide, leading to an accumulation of linearized MC-LR. The knockout of *mlrC* may reduce MlrB activity due to negative feedback from tetrapeptide accumulation. MlrD is thought to facilitate the transport of degrading products, and its deficiency may impair the ability to transport these products, causing an accumulation of linearized MC-LR in the m6-ΔmlrD group.

During the MC-LR degradation by *mlr* knockout mutants, the variations in tetrapeptide diverged from linearized MC-LR (Figure 5c). Notably, the maximum intensity of tetrapeptide in the m6-ΔmlrC group was 11.14-fold higher than that in *Sphingopyxis* sp. m6, probably attributed to the decreased conversion of the tetrapeptide to Adda. However, the tetrapeptide was further degraded, indicating that MlrC is not the sole enzyme capable of degrading the tetrapeptide. In contrast, in the m6-ΔmlrB and m6-ΔmlrD groups, the maximum intensity of the tetrapeptide decreased by 71.15% and 39.60% compared to the wild-type strain group. The blockage of MlrB, degrading linearized MC-LR to tetrapeptide in the m6-ΔmlrB group, resulted in a significant decrease in tetrapeptide levels. However, a small amount of tetrapeptide was still produced and detected. This suggests that MlrB may not be the only enzyme degrading linearized MC-LR to tetrapeptide in *Sphingopyxis* sp. m6, and that other MlrB-like enzymes with similar degrading functions may exist.

The trends of Adda during the MC-LR degradation by *mlr* knockout mutants were depicted in Figure 5d. The concentration of Adda was significantly reduced in m6-ΔmlrB, m6-ΔmlrC, and m6-ΔmlrD groups compared to *Sphingopyxis* sp. m6. The knockout of MlrC decreased (not cut off) Adda generation, which indicated that alternative pathways for Adda generation may exist in knockout mutant m6-ΔmlrC. However, the degradation of linearized MC-LR to Adda by MlrC was not observed in the wild-type strain, as the product hexapeptide compound was not detected during the degradation of MC-LR by *Sphingopyxis* sp. m6 [29]. The decrease in tetrapeptide in the m6-ΔmlrB and m6-ΔmlrD groups may contribute to the reduced formation of Adda (Figure 5c). Additionally, the impaired transport of tetrapeptide to MlrC by MlrD may further decrease Adda formation. These findings suggest that MlrD is not the sole protein responsible for the transmembrane transport of tetrapeptide in *Sphingopyxis* sp. m6.

**Figure 6:**
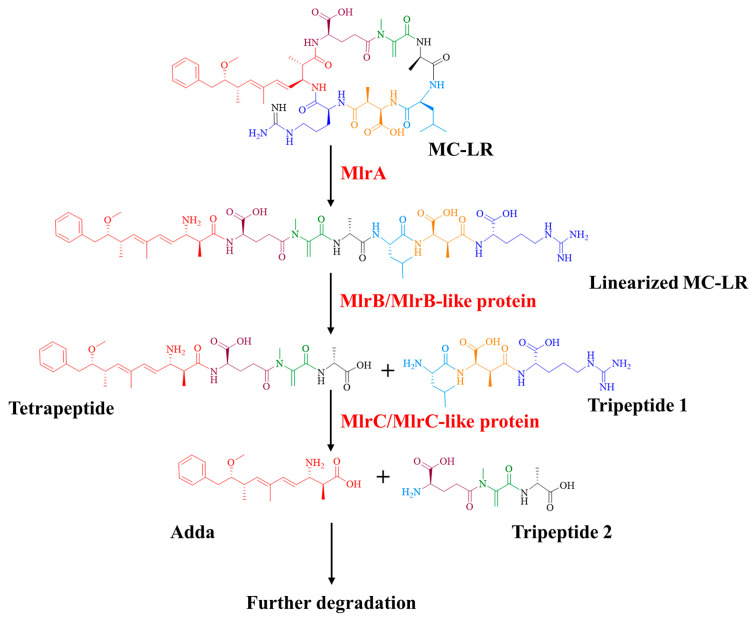
Updated degrading process of MC-LR by *Sphingopyxis* sp. m6.

According to the degrading functions of recombinant Mlr enzymes and degrading products of MC-LR by *mlr* knockout mutants, the degrading process of MC-LR by *Sphingopyxis* sp. m6 was proposed (Figure 6). In *sphingopyxis* sp. m6, MlrA cleaves MC-LR in the Arg-Adda bond into linearized MC-LR. Then, MlrB and MlrB-like enzyme cleaved the Ala-Leu bond of linearized MC-LR into tetrapeptide. MlrC and MlrC-like enzymes further degrade tetrapeptide in Adda-Glu bond to generate Adda. It should be noted that linearized MC-LR cannot be degraded by MlrC directly in *Sphingopyxis* sp. m6.

## 3. Discussion

Bacterial degradation is an important part of biodegradation for MCs removal in the natural environment [37,38,39]. *Sphingomonadaceae* is a main family among all known MC-degrading bacteria, and *Sphingopyxis* sp. m6 is a representative genus due to its high MC-degrading efficiency [14,29,31,35]. To date, the *mlr*-dependent pathway is considered to be the accepted MC-degrading mechanism [14,23,24,25,26,40]. Since 1996, Bourne et al. first identified four *mlr* genes responsible for MC-LR degradation in *Sphingomonas* sp. ACM-3962 through classic protease inhibitors and library screening [23]. They demonstrated that MlrA, MlrB, and MlrC could sequentially degrade MC-LR into linearized MC-LR, tetrapeptide, and Adda products [24]. Afterwards, Dziga and Shimizu et al. found MlrC from *Sphingomonas* ACM-3962 and *Sphingopyxis* sp. C-1 could both degrade linearized MC-LR and tetrapeptide into Adda in 2012 [25,26]. However, the characterization of Mlr enzymes and the specific MC-degrading process by Mlr enzymes in MC-degrading bacteria remains unclear. In this study, bioinformatics analysis, heterologous expression, and knockout mutagenesis of *mlr* genes were performed to elucidate the properties of Mlr enzymes and the MC-degrading mechanism.

The constrained genome distribution and conserved phylogenies of *mlr* gene cluster in *Sphingopyxis* sp. m6 suggested that these genes were inherited vertically as a functional unit within *Alphaproteobacteria* [33]. The significant difference in GC content in *mlr* gene cluster and bacterial genome indicated that *Sphingomonadaceae* acquired *mlr* gene cluster by HGT initially [41,42]. In this study, the subcellular localization of Mlr enzymes was predicted using Gneg-mPLoc algorithm, consistent with previous experimental findings [43,44]. Due to the distinct subcellular localization of Mlr enzymes, the actual MC-degrading process in the wild-type strain may differ from that in traditional heterologous expression of Mlr enzymes.

**Figure 7:**
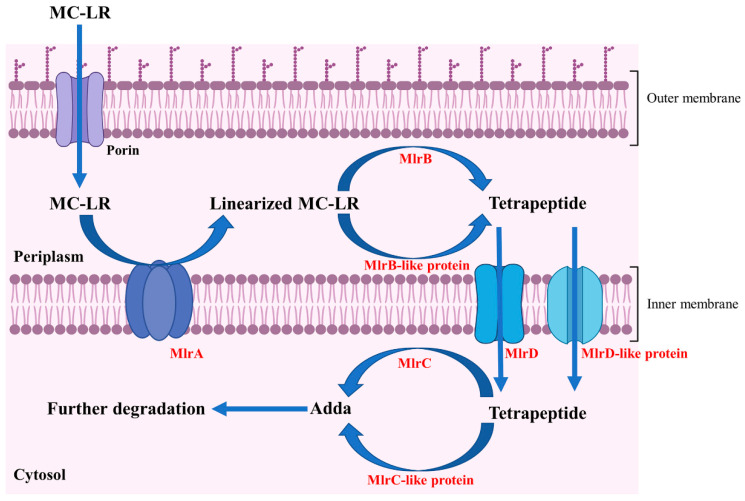
Degrading pathway of MC-LR by Mlr enzymes in *Sphingopyxis* sp. m6.

Through the heterologous expression and degrading function analysis of Mlr enzymes, we found that MlrA could open the cyclic structure of MC-LR at the Adda-Arg bond to generate linearized MC-LR. MlrB further hydrolyzes the Ala-Leu peptide bond of linearized MC-LR to produce tetrapeptide and tripeptide 1. MlrC can cleave the Adda group from the Adda-Glu peptide bond of linearized MC-LR and tetrapeptide, generating a hexapeptide compound and tripeptide 2 (Glu-Mdha-Ala), respectively (Figure S8). The degradation functions of MlrA, MlrB, and MlrC on MC-LR and its main products in *Sphingopyxis* sp. m6 are similar to those in *Sphingomonas* sp. ACM-3962, *Sphingopyxis* sp. C-1, *Sphingopyxis* sp. USTB-05, and *Sphingopyxis* sp. YF-1 [14,25,26,45,46,47,48]. Dziga et al. first identified the hexapeptide compound through the degradation of linearized MC-LR by recombinant MlrC [49]. It should be noted that the hexapeptide compound has not been previously reported in the MC-LR degradation process by wild-type MC-degrading bacteria. In this study, we also did not detect the hexapeptide compound during the MC-LR degradation by *Sphingopyxis* sp. m6 [29]. These evidences indicate that MlrB exclusively degrades linearized MC-LR, and MlrC is sole responsible for the degradation of tetrapeptide within MC-degrading bacteria (Figure 6 and Figure 7). Given that a suitable method for the functional verification of MlrD was not identified, we did not proceed with its heterologous expression and functional confirmation. Although Sun et al. ligated *mlrD* of *Sphingopyxis* sp. USTB-05 to the pET-30a vector and induced expression in *E. coli* BL21 (DE3), the function of heterologous MlrD was not verified experimentally [44]. In short, we demonstrated that recombinant MlrC degrades both linearized MC-LR and tetrapeptide in vitro, whereas its wild-type counterpart exclusively targets tetrapeptide in vivo. This critical divergence highlights the importance of studying enzymes in their native cellular context, a gap not addressed in prior work.

*Sphingopyxis* sp. m6 exhibits a high efficiency in MCs degradation, which may be attributed to the presence of multiple MC-degrading mechanisms, including *mlr*-dependent and *mlr*-independent degrading pathways [29,30,31]. Consequently, marker-free knockout mutants of MlrA, MlrB, MlrC, and MlrD were constructed to determine their individual contributions to MC-LR degradation and the existence of additional enzymes with similar degrading functions. We found that MlrA is the sole catalyst responsible for the ring-opening reaction of MC-LR, and the targeted deletion of the *mlrA* leads to a disappearance of MC-LR degradation capacity, underscoring the indispensable role of the *mlrA* in MCs degradation by *Sphingopyxis* sp. m6 (Figure 5a and Figure 6). In mutant strains m6-ΔmlrB, m6-ΔmlrC, and m6-ΔmlrD, the degrading product types of MC-LR were the same as those of *Sphingopyxis* sp. m6. However, the trends of degrading products were significantly altered (Figure 5b–d). These findings indicate that linearized MC-LR and tetrapeptide are not solely degraded by the MlrB and MlrC, but also progressively mediated by *non-mlr* enzymatic pathways (Figure 6 and Figure 7). Furthermore, MlrD-like protein exists to transmembrane transport tetrapeptide (Figure 7). As previously described, several MC-degrading bacteria can hydrolyze varieties of polypeptides in the amido bond, including MCs [50,51,52,53]. The enzymes responsible for degrading polypeptides may metabolize linearized MC-LR and tetrapeptide in mutant strains (m6-ΔmlrB and m6-ΔmlrC). It is noteworthy that the mutant strains m6-ΔmlrA, m6-ΔmlrB, m6-ΔmlrC, and m6-ΔmlrD can all degrade Adda, which further indicates that Mlr enzymes may not be involved in the Adda degradation. Maseda et al. knocked out *mlrA* in *Sphingopyxis* sp. C-1 for the first time, discovering that MlrA is the only enzyme responsible for initiating the degradation of cyclic MCs through MC-LR degradation tests [43]. However, the catechol 2,3-dioxygenase gene (*xylE*) and kanamycin resistance gene (*KmR*) were introduced as selection and indicator markers [43]. In this study, *mlrA* was deleted without any insertion of an exogenous gene. In addition, we have not found any studies that constructed *mlrB*, *mlrC*, and *mlrD* knockouts and performed functional analyses to date. To the best of our knowledge, our study is the first to achieve marker-free knockout of *mlrA*, *mlrB*, *mlrC*, and *mlrD* in *Sphingopyxis* sp. m6, enabling functional analysis without genetic interference. This revealed compensatory mechanisms via MlrB-/MlrC-/MlrD-like enzymes, a novel finding in MC degradation research.

Combining the subcellular localization, degrading function of recombinant Mlr enzymes, and degrading products of MC-LR by Mlr knockout mutants, a novel MC-LR degrading pathway by Mlr enzymes in *Sphingopyxis* sp. m6 was proposed (Figure 7). During the degradation of MC-LR by *Sphingopyxis* sp. m6, MC-LR initially traverses the outer membrane and enters the periplasm, where it binds to MlrA located on the inner membrane. MlrA cleaves the Adda-Arg bond of MC-LR and generate linearized MC-LR. Subsequently, linearized MC-LR is released into the periplasm and binds to MlrB, leading to the formation of tetrapeptide. Concurrently, it is possible that a portion of linearized MC-LR is degraded into tetrapeptide by MlrB-like enzymes. Thereafter, MlrD transports tetrapeptide across the inter membrane into the cytoplasm. In the cytoplasm, MlrC cleaves the Adda-Glu bond in tetrapeptide and produce Adda. Additionally, other enzymes functionally similar to MlrC (MlrC-like) may contribute to the degradation of tetrapeptide into Adda. Finally, Adda is further metabolized and degraded in *Sphingopyxis* sp. m6. Previous studies predicted that MlrD is responsible for the transport of MCs and their degrading products by analyzing the conserved motif in the amino acid sequence [24]. In this study, we predicted that MlrD is mainly responsible for the transmembrane transport of tetrapeptides based on the degradation process of MC-LR by *Sphingopyxis* sp. m6. However, all these speculations need to be verified by further functional experiments. In summary, this study uniquely combines bioinformatics, heterologous expression, and knockout mutagenesis to propose a revised degradation pathway (Figure 6 and Figure 7), incorporating subcellular localization and *non-mlr* enzymatic synergies—advancing beyond the traditional in vitro paradigm.

## 4. Conclusions

MCs pose a serious threat to ecological environmental safety and public health due to the ubiquitous contamination and potent toxicity. Bacterial degradation represents as one important MCs removal method in the natural environment. Bacterial degradation by Mlr enzymes is currently recognized as the predominant MC-degrading pathway. However, the MC-degrading mechanism by Mlr enzymes in wild-type bacteria is unclear. In this study, *Sphingopyxis* sp. m6, an efficient MC-degrading bacteria and harboring Mlr enzymes, was used as a representative to analyze the specific degrading mechanism. We found that the *mlr* gene cluster is located on the same genomic island of the genome, which initially derived from HGT, and then maintained vertical inheritance. Mlr enzymes were first knocked out from *Sphingopyxis* sp. m6 and a novel MC-degrading pathway was proposed for the wild-type strain. During the degradation of MC-LR by *Sphingopyxis* sp. m6, MlrA hydrolyze cyclic MC-LR to linearized MC-LR. MlrB degrades linearized MC-LR into a tetrapeptide and a tripeptide. MlrC further decomposes the tetrapeptide into adda. To be noted, MlrA is the solo enzyme responsible for the first step of MC-LR degradation. The other genes with similar functions to *mlrB*, *mlrC*, and *mlrD* may exist in *Sphingopyxis* sp. m6, which collaboratively contribute to the sequential degradation of linearized MC-LR into adda. In addition, unlike dual degradation functions (linearized MC-LR and tetrapeptide) of MlrC demonstrated in vitro, it exclusively degrades tetrapeptide intracellularly in MC-degrading bacteria. This study elucidates the MC-degrading mechanism by Mlr enzymes in *Sphingopyxis* sp. m6 and proposes a potential research direction for exploring the bacterial degrading mechanism.

## 5. Materials and Methods

### 5.1. Bacterial Strains and Reagents

The MC-degrading bacterium *Sphingopyxis* sp. m6 served as the genomic template for *mlr* cluster amplification [29]. Competent cells *E. coli* DH5α and *E. coli* BL21 (DE3) were purchased from Vazyme Biotech Co., Ltd. (Nanjing, China). Suicide T-vectors pLP12 and *E. coli* β2163 were provided by KnoGen Biotech Co., Ltd. (Guangzhou, China). The HMBP-pET-28a expression vector was obtained by Jingzhun Biotech Co., Ltd. (Nanjing, China).

Standard MC-LR (≥95%) was acquired from Enzo Life Sciences Incorporation (Farmingdale, NY, USA). High performance liquid chromatography (HPLC) grade solvents (methanol, acetonitrile) and trifluoroacetic acid were purchased from Merck (Darmstadt, Germany) and TCI Chemicals (Shanghai, China), respectively. Molecular biology reagents, including restriction enzymes (NdeI, XhoI), T4 DNA ligase, PrimeSTAR Max DNA polymerase, and Exnase II (ClonExpress II), were acquired from TransGen Biotech (Beijing, China), TaKaRa Bio (Beijing, China), and Vazyme (Nanjing, China). Kanamycin and chloramphenicol (Sangon Biotech, Shanghai, China), 2,6-diaminopimelic acid (DAP, Macklin, Shanghai, China), and formic acid (Fisher Scientific, Shanghai, China) were also used in this study. Detailed preparation protocols for mineral salt medium (MSM) followed our established methodology [29].

### 5.2. Bioinformatic Analysis of Mlr Enzymes

The distribution and completed sequences of the *mlr* gene cluster in *Sphingopyxis* sp. m6 were identified using the NCBI Nucleotide BLAST (https://blast.ncbi.nlm.nih.gov/Blast.cgi, accessed on 2 July 2025). Phylogenetic reconstruction employed ClustalW-aligned sequences and neighbor-joining algorithms in MEGA-X. Transmembrane domains were predicted via TMHMM v2.0 and DeepTMHMM, while SignalP-5.0 analyzed signal peptides. Physicochemical properties of Mlr enzymes were calculated using ExPASy ProtParam, with subcellular localization predicted by Gneg-mPLoc. Protein motifs and conserved structural domains were identified using the CD-search program of the NCBI database.

### 5.3. Heterologous Expression of Mlr Enzymes

The *mlrA*, *mlrB,* and *mlrC* genes were amplified from the *Sphingopyxis* sp. m6 genome using the primers (Table S2) and further purified from an agarose gel. Restriction endonucleases (NdeI and XhoI) were used to cleave the HMBP-pET-28a vector and *mlrA*, *mlrB,* and *mlrC* amplification products. Then, the products and the vector were ligated by T4 DNA ligase to construct recombinant plasmids HMBP-pET28a-mlrA, HMBP-pET28a-mlrB, and HMBP-pET28a-mlrC. The ligated products were transformed into *E. coli* DH5α and further verified through sequencing. The correct recombinant plasmids were selected and transformed into *E. coli* BL21 (DE3). Finally, the recombinant strains harboring *mlrA*, *mlrB,* and *mlrC* genes were induced by IPTG, and their encoded proteins were analyzed by SDS-PAGE. Notably, *mlrD* was excluded due to challenges in the functional validation of its transmembrane transporter.

### 5.4. Protein Purification and Enzymatic Activity

The Mlr enzymes were further purified to test their degrading ability on MC-LR and its degrading products according to a previous study [36]. Maltose-binding protein (MBP)-tagged Mlr enzymes were affinity-purified under native conditions using Ni-NTA chromatography (Tiandz, Beijing, China) following the manufacturer’s protocol. Protein concentrations were quantified via Bradford assay (Bio-Rad). Reaction systems containing 1 mg/L MC-LR or its major degrading products and 0.05 mg/L purified enzymes (substrate-to-enzyme ratio 9:1 *v*/*v*) were incubated at 30 °C (180 rpm, dark). Aliquots collected at predetermined intervals were immediately heat-inactivated (95 °C, 5 min) to terminate enzymatic activity, and the residual degrading substates were further quantitated. Additionally, an equal volume of heat-inactivated enzymes to replace purified enzymes was used as a control.

### 5.5. Markerless Knockout Mutant of mlr Genes

MC-degrading genes (*mlrA*, *mlrB*, *mlrC*, and *mlrD* genes) of *Sphingopyxis* sp. m6 were deleted through double homologous recombination between *Sphingopxis* sp. m6 and *E. coli* β2163 harboring suicide vector pLP12 (GenBank: KT326153.1) (Tables S3–S6). Vector pLP12 has the CM resistance gene, and *E. coli* β2163 is DAP nutritionally deficient. The upstream and downstream fragments of the target genes were cloned into pLP12 to construct recombinant suicide vectors. Transconjugants were selected on MSM agar supplemented with 30 μg/mL CM and 0.3 mM DAP. Mutants were validated through antibiotic sensitivity profiling and Sanger sequencing of target genes.

### 5.6. Degrading Function of Knockout Mutants

The constructed mutants were inoculated into LB medium and cultivated to the exponential growth phase (30 °C, 180 rpm). Then, the cells were harvested and washed twice with sterile phosphate-buffered saline (PBS). Subsequently, the pellets were resuspended in ultrapure water to OD_600_ = 1 and inoculated into MSM (*v*/*v* = 1/9), with a final concentration of 1 mg/L MC-LR. The cultures were incubated at 30 °C with a shaking speed of 180 rpm in the dark. Samples were collected at designated intervals and centrifuged to analyze the residual MC-LR and metabolites in supernatants [29].

### 5.7. Determination of MC-LR and Degrading Products

High-performance liquid chromatography (HPLC, Agilent, Santa Clara, CA, USA) and a Zorbax Extend C18 column (1.8 µm, 2.1 × 50 mm, Agilent, USA) was used to determine the concentration of MC-LR [29]. Meanwhile, ultra-performance liquid chromatography coupled with a tandem mass spectrometer system (UPLC-MS/MS, AB SCIEX, Redwood, CA, USA) equipped with a Waters ACQUITY HSS T3 column (1.8 μm, 2.1 × 100 mm) was performed in IDA (information-dependent analysis) mode to identify the degrading products of MC-LR. Further, HPLC was used to detect the concentrations of major degrading products in the recombinant enzyme analysis. Due to the detection limit of HPLC, the concentrations of degrading products were determined using UPLC-MS/MS equipped with an ACQUITY BEH C18 (1.7 μm, 2.1 × 50 mm, waters, Millford, MA, USA) in the knockout mutant analysis. The specific parameters are detailed in our previous publication [18].

### 5.8. Statistical Analysis

In this study, the experiments were performed with three independent replicates, and the data were shown as mean ± SD (standard deviation). One-way ANOVA was performed to determine statistical differences. *p* ≤ 0.05 was considered to be statistically significant.

## Figures and Tables

**Table 1:** Structural attributes of Mlr enzymes.

Enzyme	Signal Peptide	TM Helices	pI	MW (Da)	Subcellular Localization	Conserved Catalytic Domain
MlrA	Y	8	9.32	36, 317.07	Cell inner membrane	Type II CAAX prenyl endopeptidase Rce1-like
MlrB	Y	0	6.78	59, 075.15	Periplasm	Serine hydrolase domain-containing protein
MlrC	N	0	7.06	57, 152.61	Cytoplasm	M81 family metallopeptidase
MlrD	N	12	9.19	45, 001.83	Cell inner membrane	MFS transporter, dipeptide/tripeptide permease

Y: Yes; N: No.

## Data Availability

The original contributions presented in this study are included in the article/Supplementary Material. Further inquiries can be directed to the corresponding authors.

## Supplementary Materials

The following supporting information can be downloaded at: https://www.mdpi.com/article/10.3390/toxins17090446/s1, Figure S1. Flow chart of the experiment procedures; Figure S2. Phylogenetic relationship of the *mlrA* gene in *Sphingopyxis* sp. m6; Figure S3. Phylogenetic relationship of the *mlrB* gene in *Sphingopyxis* sp. m6; Figure S4. Phylogenetic relationship of the *mlrC* gene in *Sphingopyxis* sp. m6; Figure S5. Phylogenetic relationship of the *mlrD* gene in *Sphingopyxis* sp. m6; Figure S6. Prediction of transmembrane regions of MlrA; Figure S7. Prediction of transmembrane regions of MlrD; Figure S8. Alignment of the amino acid sequence of MlrA with its encoding gene sequence; Figure S9. Alignment of the amino acid sequence of MlrB with its encoding gene sequence; Table S1. Degrading ability of Mlr enzymes on MC-LR and its main products in *Sphingopyxis* sp. m6; Table S2. Primers used to construct the heterologous expression system of *mlr* gene cluster; Table S3. Primers used in the construction of the *mlrA* knockout mutant of *Sphingopyxis* sp. m6; Table S4. Primers used in the construction of the *mlrB* knockout mutant of *Sphingopyxis* sp. m6; Table S5. Primers used in the construction of the *mlrC* knockout mutant of *Sphingopyxis* sp. m6; Table S6. Primers used in the construction of the *mlrD* knockout mutant of *Sphingopyxis* sp. m6.
