# Supplementary material for: Comprehensive Insight into Microcystin-Degrading Mechanism of Sphingopyxis sp. m6 Based on Mlr Enzymes
Source: Toxins (Basel). 2025 Sep 5;17(9):446. doi: 10.3390/toxins17090446 (PMC12474180; doi:10.3390/toxins17090446)
Supplement: Supplementary file 1 [file toxins-17-00446-s001.zip › toxins-3766231-supplementary.pdf]

Supplementary Materials: Comprehensive Insight into Microcystin-Degrading Mechanism of *Sphingopyxis* sp. m6 Based on Mlr Enzymes

Qin Ding, Tongtong Liu, Zhuoxiao Li, Rongli Sun, Juan Zhang *, Lihong Yin * and Yuepu Pu

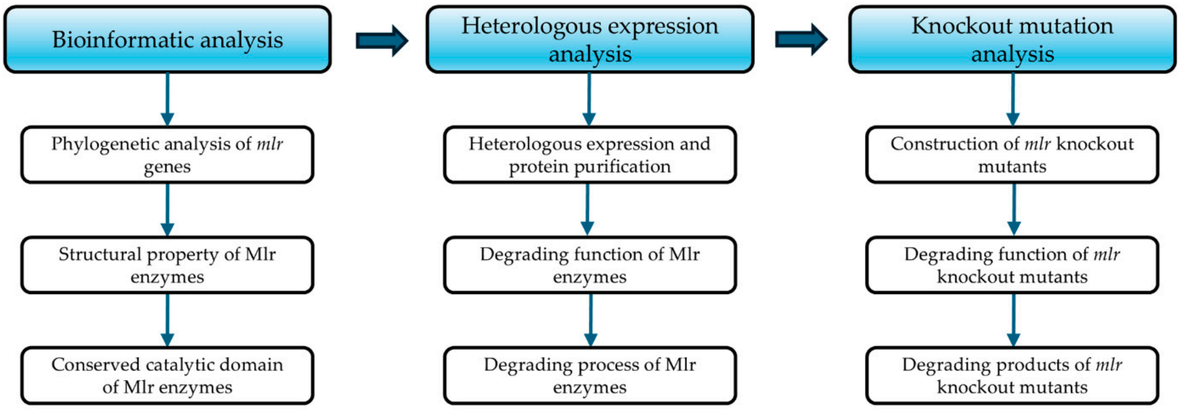

Figure S1. Flow chart of the experiment procedures.

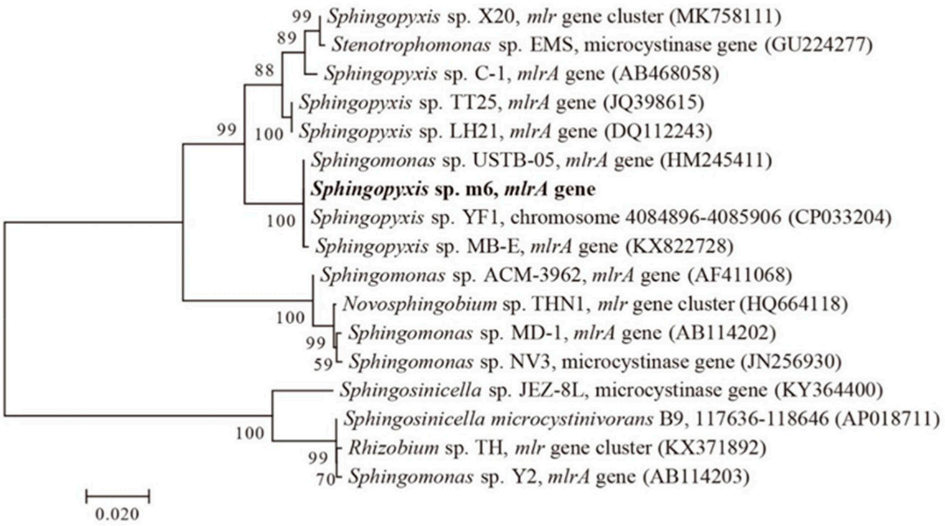

Figure S2. Phylogenetic relationship of *mlrA* gene in *Sphingopyxis* sp. m6.

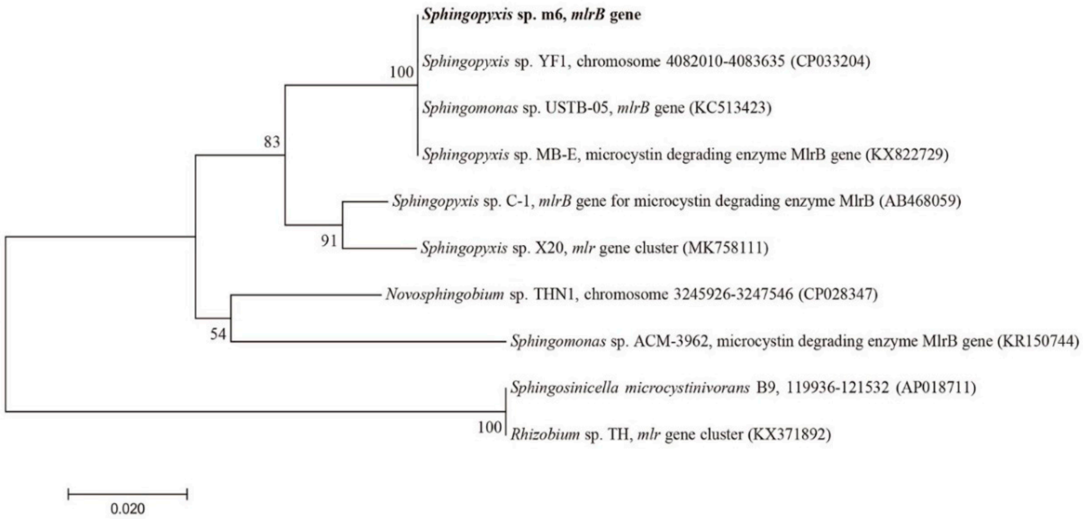

Figure S3. Phylogenetic relationship of *mlrB* gene in *Sphingopyxis* sp. m6.

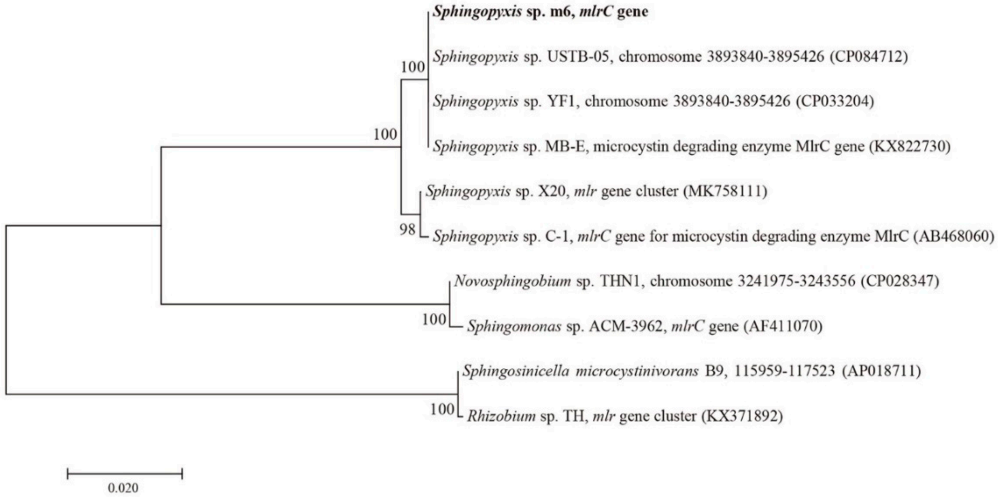

Figure S4. Phylogenetic relationship of *mlrC* gene in *Sphingopyxis* sp. m6.

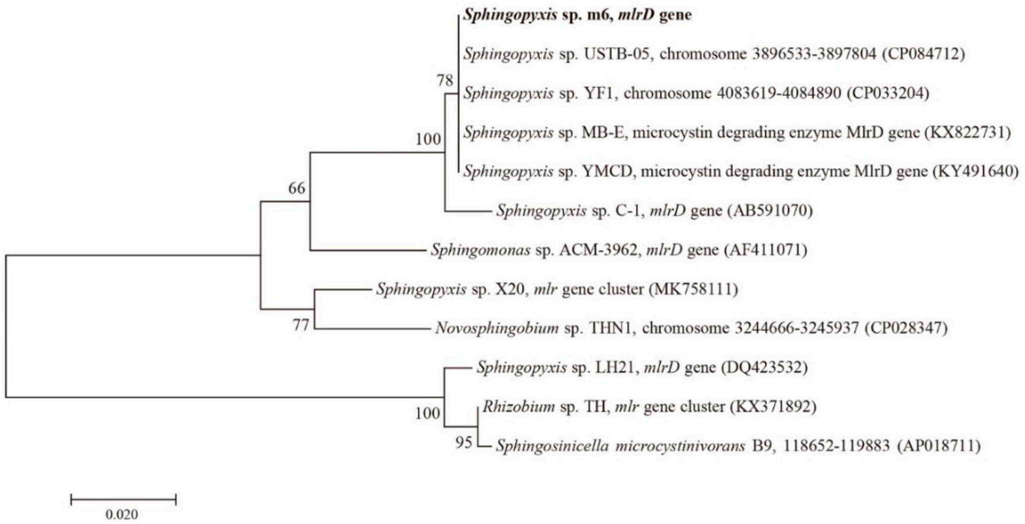

Figure S5. Phylogenetic relationship of *mlrD* gene in *Sphingopyxis* sp. m6.

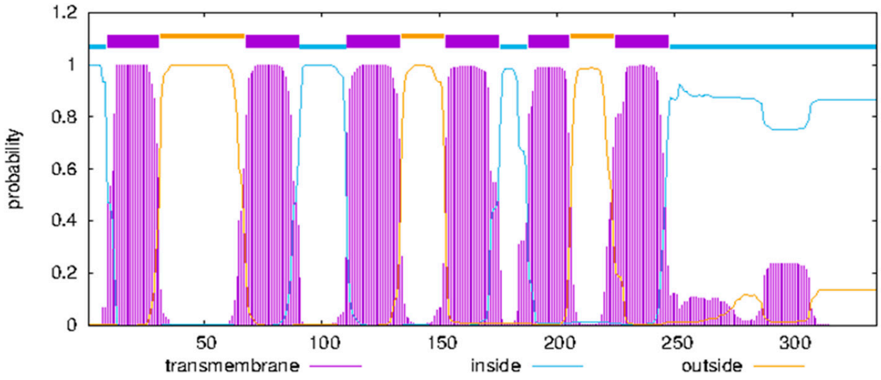

Figure S6. Prediction of transmembrane regions of MlrA.

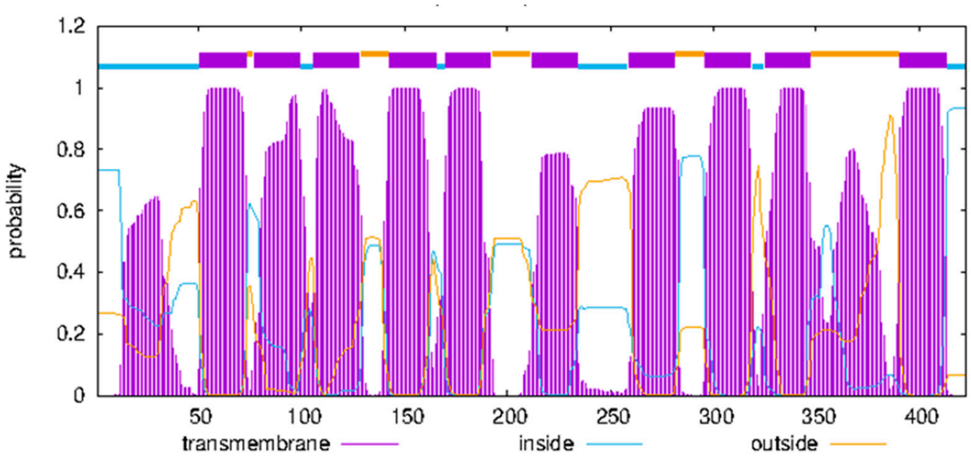

Figure S7. Prediction of transmembrane regions of MlrD.

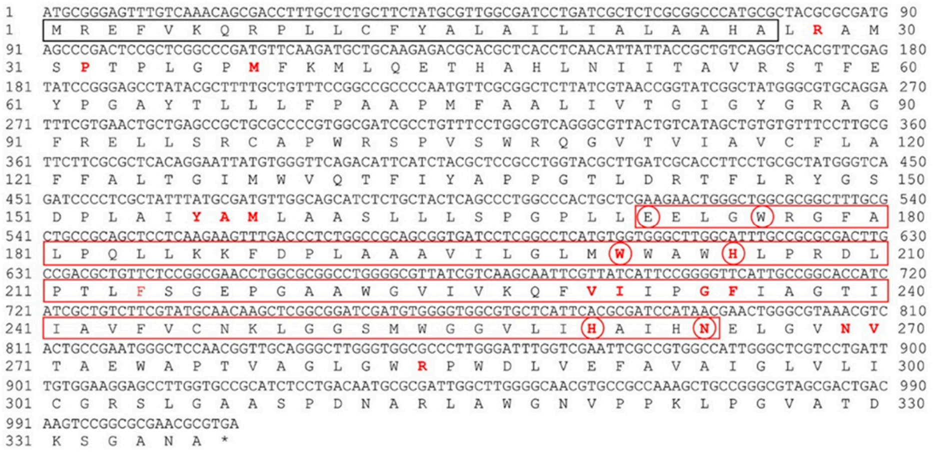

Figure S8. Alignment of the amino acid sequence of MlrA with its encoding gene sequence.
(The black box and red box represent the signal peptide sequence and the Abi domain, respectively. The red bold font indicates the binding sites between MlrA and MC-LR. The red circle highlights the conserved amino acid residue sites of the Abi domain.)

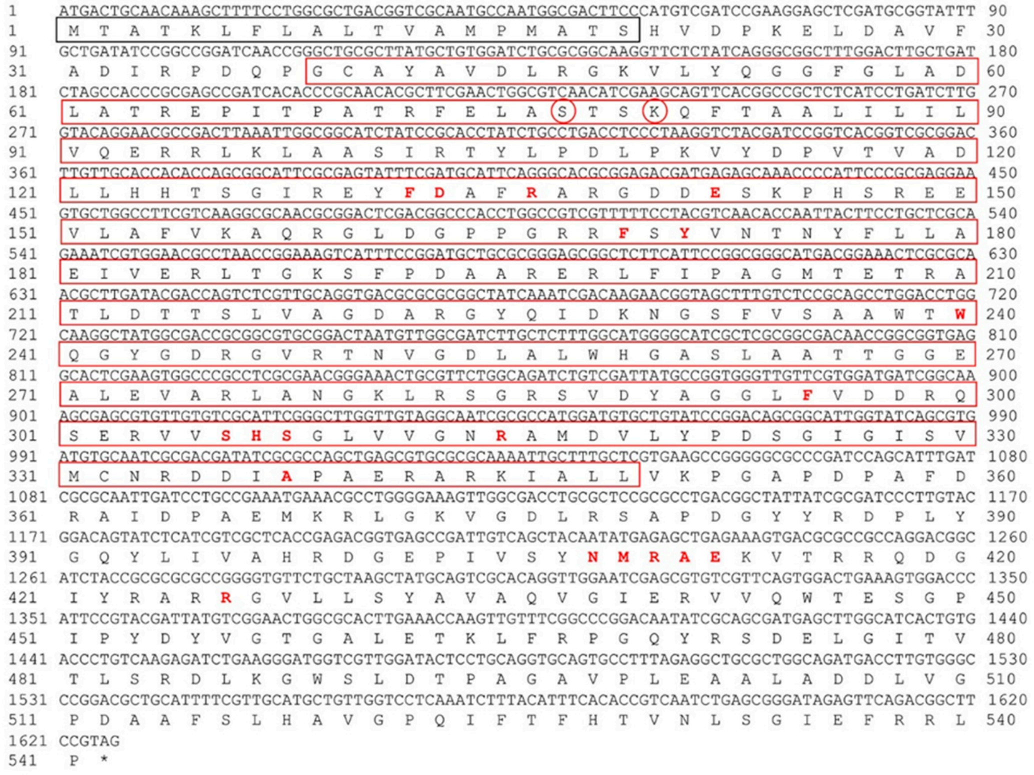

Figure S9. Alignment of the amino acid sequence of MlrB with its encoding gene sequence.
(The black box and red box represent the signal peptide sequence and the Abi domain, respectively. The red bold font indicates the binding sites between MlrA and MC-LR. The red circle highlights the conserved amino acid residue sites of the Abi domain.)

Table S1. Degrading ability of Mlr enzymes on MC-LR and its main products in Sphingopyxis sp. m6

	MC-LR	Linearized MC-LR	Tetrapeptide	Adda
MlrA	+	-	-	-
MlrB	-	+	-	-
MlrC	-	+	+	-

+: can degrade. -: can't degrade.

Table S2. Primers used to construct the heterologous expression system of *mlr* gene cluster.

Target gene	Primer name	Sequence（5'-3'）
mlrA	mlrA-NdeI-F	GGGAATTC *CATATG* CGGGAGTTTGTCAAACAGCGACCTTTGC
	mlrA-XhoI-R	CCG *CTCGAG* TCACGCGTTCGCGCCGGACTTGTCAGTCGCTACGCCCGGC AGCTTTGGC
mlrB	mlrB-NdeI-F	GGGAATTC *CATATG* ACTGCAACAAAGCTTTTCCTGGCGCTGACGGTCGC
	mlrB-XhoI-R	CCG *CTCGAG* CTACGGAAGCCGTCTGAACTCTATCCCGC
mlrC	mlrC-NdeI-F	GGGAATTC *CATATG* GCAACCCTGCTGGGTCGAGGGTTG
	mlrC-NotI-R	AAGGAAAAAA *GCGGCCGC* CTAGGCTGAAAAGTCGACAGGCTCGAATGGCCAC

Table S3. Primers used in the construction of *mlrA* knockout mutant of *Sphingopyxis* sp. m6

Primer	Sequence (5' - 3')
mlrA-MF1	GGAATCTAGACCTTGAGTCG TGTAAACCGAAAGCCACGAT
mlrA -MR1	ATTGTCAGGAGATGCGGCACCA GCCAACGCATAGAAGCAGA
mlrA -MF2	TCTGCTTCTATGCGTTGGC TGGTGCCGCATCTCCTGACAA
mlrA -MR2	ACAGCTAGCGACGATATGTC GTAGCGGAAGCGAGGAAGCTGTA
mlrA -TF	GCGACAAGTGAGCGTGAAGA
mlrA -TR	CGGCGAGAAAGATAACGATG
pLP-UF	GACACAGTTGTAACTGGTCCA
pLP-UR	CAGGAACACTTAACGGCTGAC

Table S4. Primers used in the construction of *mlrB* knockout mutant of *Sphingopyxis* sp. m6

Primer	Sequence (5' - 3')
mlrB-TF	GACGCCATCTTCTGCCTCCA
mlrB-MF1	GGAATCTAGACCTTGAGTCG CGGAAGCCGTCTGAACTCTATC
mlrB-MR1	ATGAGCGAGGCAAGATCTG ATGATGAAACGCCTGGGGAAAGT
mlrB-MF2	ACTTTCCCCAGGCGTTTCATCAT CAGATCTTGCCTCGCTCAT
mlrB-MR2	ACAGCTAGCGACGATATGTC ATCTTCTCGGCGTCGTCTGC

Table S5. Primers used in the construction of *mlrC* knockout mutant of *Sphingopyxis* sp. m6

Primer	Sequence (5' - 3')
mlrC-TF	GGTACGGGATTGCTGCTAGTTC
mlrC-TR	ACGAAGACAGCGATGATGGTG
mlrC-MF1	GGAATCTAGACCTTGAGTCG TCACTGCCCCTGTTCCAAGA
mlrC-MR1	CGAACAACATCAAACCGAAGACT TCAGCCTAGCGAATAAACCAGAC
mlrC-MF2	GTCTGGTTTATTCGCTAGGCTGA AGTCTTCGGTTTGATGTTGTTCG
mlrC-MR2	ACAGCTAGCGACGATATGTC GCTGCCAACATCGCATAAATAG

Table S6. Primers used in the construction of *mlrD* knockout mutant of *Sphingopyxis* sp. m6

Primer	Sequence (5' - 3')
mlrD-TF	TTACCGCTGTCAGGTCCACG
mlrD-TR	ACAGCGGCATTGGTATCAGC
mlrD-MF1	GGAATCTAGACCTTGAGTCG TTCAGACATTCATCTACGCTCCG
mlrD-MR1	AAGAAGTTGCCATATCCGAGTGC TTCCGTCGCAGCCAGCAGTA
mlrD-MF2	TACTGCTGGCTGCGACGGAA GCACTCGGATATGGCAACTTCTT
mlrD-MR2	ACAGCTAGCGACGATATGTC AATGAAACGCCTGGGGAAAG
